# Supplementary material for: Time to incidence of tuberculosis and its predictors among adult HIV/AIDS patients who initiated ART by the Universal Test and Treat approach in Silte Zone, Ethiopia, 2023
Source: PLOS Glob Public Health. 2026 Apr 10;6(4):e0005970. doi: 10.1371/journal.pgph.0005970 (PMC13068246; doi:10.1371/journal.pgph.0005970)
Supplement: S1 Text — (DOCX) [file pgph.0005970.s001.docx]

**Additional methods and results**

Adult HIV patients enrolled to HIV care by universal test and treat approach in Silte Zone public health facilities (1,257)

WCSH

141

WHC

212

Tora PH

72

Lera HC 203

Mugo

HC 81

AGPH

44

Kibet PH 62

Shilimat HC

49

Kilto HC 72

Adazer HC 55

Kowakoto HC 115

Hulbareg HC 34

Dalocha HC 89

50

17

75

40

19

25

70

25

28

12

16

31

Proportional allocation with SRS

**442**

Mito HC

28

10

22

*Fig A. Sampling procedure to assess incidence of tuberculosis and its predictors among patients on HAART with the UTT approach, 2022.*

Table A. Log-rank test for equality of survivor functions between categories of covariates

| Variables | Chi 2 (X2) | Pr > chi2 |
| --- | --- | --- |
| Age | 6.74 | 0.0807 |
| Sex | 1.57 | 0.2075 |
| Catchment area | 3.82 | 0.0506 |
| Nutritional status | 16.52 | 0.0003 |
| Disclosure | 16.21 | <0.001 |
| Opportunistic infection other than Tuberculosis | 4.93 | 0.0265 |
| Non-AIDS related chronic disease | 4.15 | 0.0416 |
| IPT | 2.59 | 0.1078 |
| WHO clinical stage | 41.40 | <0.001 |
| Adherence | 79.23 | <0.001 |
| Functional status | 52.93 | <0.001 |
| CD-4 count | 52.17 | <0.001 |
