# Supplementary material for: Time to incidence of tuberculosis and its predictors among adult HIV/AIDS patients who initiated ART by the Universal Test and Treat approach in Silte Zone, Ethiopia, 2023
Source: PLOS Glob Public Health. 2026 Apr 10;6(4):e0005970. doi: 10.1371/journal.pgph.0005970 (PMC13068246; doi:10.1371/journal.pgph.0005970)
Supplement: Attachment — Submitted filename: Response to Reviewers.docx [file pgph.0005970.s004.docx]

**Response to Reviewers**

Dear Damen Haile Mariam, MD, MPH, PhD,
Academic Editor, *PLOS Global Public Health*,

Dear Editors and Reviewers,

We sincerely appreciate the time and effort devoted to the evaluation of our manuscript and thank you for the insightful and constructive comments provided. In response, we have carefully revised the manuscript to ensure full compliance with *PLOS Global Public Health* journal requirements and have prepared a detailed, point-by-point response addressing each comment raised by the reviewers.

All revisions have been incorporated into the manuscript and are highlighted in yellow in the document labeled “Revised Manuscript with Track Changes,” with corresponding sections clearly indicated to facilitate review. In addition, an unmarked version of the revised manuscript without tracked changes has been uploaded as a separate file labeled “Manuscript.”

We trust that these revisions adequately address the reviewers’ concerns and enhance the overall quality, clarity, and scientific rigor of the work. We are grateful for the opportunity to revise and resubmit our manuscript for your consideration.

Kind regards,
The Authors

**Journal Requirements:**

**1. Please ensure that your Ethics Statement is available in its entirety at the beginning of your Methods section, under a subheading 'Ethics Statement'.**

**Author's Response**: Thank you for the suggestion. As recommended, the ethical statement is now presented in full at the beginning of the Methods section of the revised manuscript.

**2. Please upload separate figure files in .tif or .eps format. Also, remove the figures from your manuscript file but keep the legends.**

**Author's Response**: Thank you for your constructive recommendation. To ensure that all figures in the submission matched PLOS technical standards, the PACE tool was used (https://pacev2.apexcovantage.com/). In accordance with the journal's rules, all figures have been uploaded as independent files in TIF format. The figures have been removed from the main manuscript file, while the corresponding figure legends have been retained in the manuscript, as recommended.

**3. We have noticed that you have cited Tables 1 to 8 in the manuscript file but there are no corresponding tables in the manuscript. Please amend your manuscript to include this table, noting that tables should not be uploaded as individual files.**

**Author's Response:** Thank you for this comment. All tables cited from Table 1 through Table 8 have now been appropriately included in the Results section of the revised manuscript and placed immediately after their respective captions, as suggested.

In response to the reviewers’ recommendation to reduce the number of tables, several tables were merged. Specifically, Tables 2 (“Behavioral characteristics of patients enrolled on HAART with the UTT approach, 2023”), Table 3 (“Clinical and immunological characteristics of patients enrolled on HAART with the UTT approach, 2023”), and Table 4 (“Medication-related characteristics of patients enrolled on HAART with the UTT approach, 2023”) in the previous manuscript version have been compiled into a single table. This consolidated table is now presented as Table 2, titled “Behavioral, Clinical, Immunological, and Medication-Related Characteristics of Patients Enrolled in HAART under the UTT Approach, 2023,” in the revised manuscript.

Additionally, some tables have been removed from the main manuscript and provided as Supporting Information as supplementary material, in accordance with the reviewers’ recommendations.

**4. We have noticed that you have uploaded Supporting Information files, but you have not included a list of legends. Please add a full list of legends for your Supporting Information files after the references list.**

**Author's Response:** Thank you for the comment. As per your suggestion, we have included a complete list of legends for all Supporting Information files immediately after the References section in the revised manuscript as follows.

Supporting information file 1 (S 1): TB-HIV STATA dataset

**5. In the online submission form, you indicated that “All relevant materials and data are within the manuscript and its supporting files and if any additional data or clarification needed, will be available from the corresponding author upon reasonable request.”.**

**Author's Response:** We appreciate the comment regarding the PLOS ONE data availability policy. In full compliance with this requirement, we confirm that all data underlying the findings reported in this manuscript have been made freely and openly accessible. The complete dataset supporting the results and conclusions of the study is provided within the manuscript and its Supporting Information files. By making the data available in this manner, we ensure transparency, facilitate independent verification, and allow other researchers to build upon the results of the present study, in accordance with PLOS ONE’s commitment to open and reproducible research.

**Response to Reviewer 1**

**General Comments**

**1. The document requires- serious editorial revisions. For instance, the word "taste" is used in the place of "test" in the title of the manuscript.**

**Author's Response**: We appreciate the reviewer's insightful feedback. As stated, extensive editing changes have been made to the entire document.

**2. The authors have not made distinction between TB disease and TB infection (Refer to the WHO criteria).**

**Author's Response:** We thank the reviewer for this insightful and constructive comment and fully agree that it represents an important correction that improves the clarity and scientific accuracy of the manuscript. According to the World Health Organization, tuberculosis (TB) is classified into two states: TB infection and TB disease. TB infection (formerly latent or inactive TB) refers to the presence of TB bacteria without symptoms or risk of transmission, although progression to active disease is possible without treatment. TB disease occurs when the immune system fails to contain the bacteria, resulting in active replication, clinical symptoms, and potential transmissibility. As the focus of our study is on active TB disease, we have revised the manuscript accordingly by replacing references to TB infection with TB disease. These revisions and corrections have been implemented consistently throughout the entire manuscript, as recommended.

**3. There is lot of redundancy; thus it requires diligent editorial work.**

**Author's Response:** We are grateful to the reviewer for this significant observation. The book has been carefully and diligently edited to remove redundancy across the text. Repetitive statements have been deleted or condensed, sentences have been refined, and overlapping content has been updated to increase clarity, conciseness, and general readability while maintaining the work's scientific purpose.

**4. Abstract - The results should start with describing the cohort characteristics.**

**Author's Response:** We thank the reviewer for the insightful comment. The Results section of the abstract has been revised accordingly and is presented as follows.

**Result**: Among 404 HIV-positive adults, the median age was 36 years with near-equal sex distribution. Most patients were in WHO stages I–II at ART initiation, about 70% showed good adherence, over 80% disclosed their HIV status, and TDF-3TC-EFV was the predominant regimen. The overall incidence density rate (IDR) of tuberculosis was 5.33 per 1000 person-months of observation (95% CI: 3.68–7.77). The incidence of new infection was 4.0 per 1000 person-months (95% CI: 2.60–6.13), while the incidence of reinfection was 1.3 per 1000 person-months (95% CI: 0.63–2.70). The survival probabilities at the 6th, 12th, and 18th months of follow-up were 0.99, 0.93, 0.90, and 0.87, respectively. Factors significantly associated with increased risk of tuberculosis included male sex [AHR: 5.05, 95% CI: 1.79–14.28], no disclosure of HIV status [AHR: 6.29, 95% CI: 1.96–20.19], being underweight [AHR: 3.07, 95% CI: 1.23–7.65], CD4 count <200 cells/μL [AHR: 5.63, 95% CI: 1.72–18.38], and poor ART adherence [AHR: 7.05 95% CI: 1.99-25.01].

**5. Methods - What is the power of the study?**

**Author's Response**: Thank you for raising this important point. Although the study eventually used a survival analysis to estimate incidence and identify predictors, the sample size was determined using a single population proportion (SPP) formula because the cohort was initially designed to estimate the overall incidence of tuberculosis among HIV-positive people rather than to test a specific hypothesis or compare groups. As a result, the SPP approach was employed to assure acceptable precision of the incidence estimate based on a predetermined confidence level and margin of error, rather than explicit statistical power. Nonetheless, the final sample size and number of events observed were sufficient to support the survival analysis, and the accuracy of the Hazard Ratio estimates and their Confidence Interval show the sample's suitability for identifying significant variables.

Power, defined as the chance of detecting a true effect (1−β), is commonly used in hypothesis-testing or comparison studies. However, for descriptive investigations employing a single population proportion (SPP) technique, sample size is determined using estimating precision rather than hypothesis testing. As a result, the SPP method does not explicitly contain a power term; rather, the sample size is calculated by the confidence level, predicted proportion, and margin of error. Under large-sample situations, these values implicitly assure good statistical reliability, and commonly used assumptions (e.g., 95% confidence level, 5% margin of error, and P = 0.5) give a conservative sample size that is typically sufficient for exact estimation and acceptable power.

**6. Results - What proportion of patients were given TB preventive therapy? This is part of the care bundle. Most received tenofovir, lamivudine and efavirenz, but nationally it is tenofovir, lamivudine and dolutegravir? Is the reason known?**

**Author response:** We thank the reviewer for this insightful and important comment. In our study, approximately 70% of the participants received tuberculosis preventive therapy (IPT) as part of the standard HIV care bundle.

With regard to antiretroviral therapy, although Ethiopia nationally adopted the tenofovir/lamivudine/dolutegravir (TLD) regimen as the preferred first-line ART following updates to the national HIV prevention, care, and treatment guidelines in 2019, most participants in this cohort were initially initiated on tenofovir/lamivudine/efavirenz-based regimens. This discrepancy is explained by the timing of ART initiation among the study population. Ethiopia began implementing the Universal Test and Treat (UTT) strategy nationally in August 2016, and the ART regimens reported in this analysis correspond to the regimen prescribed at the time of enrollment into ART care.

As a result, the ART regimen data captured in this study reflect baseline treatment rather than subsequent regimen modifications. Many patients may have later transitioned to TLD following national scale-up; however, regimen switches during follow-up were not systematically captured and therefore are not reflected in the reported ART distribution. We have clarified this point in the manuscript to avoid misinterpretation and to better align the findings with national ART policy evolution.

**7. Discussion - What are the study limitations?**

**Author response:** We appreciate the reviewer for this constructive question. In response, we have added a concise description of the study limitations in the Discussion section, and these revisions have been incorporated into the revised version of the manuscript.

**Study limitations:**

There are a number of limitations to this study. Its retrospective design depended on routinely gathered clinical records, which could be misclassified and had inadequate documentation. Discrepancies with current treatment guidelines may be explained by the fact that ART regimens were recorded at the time of ART initiation and did not capture later regimen switches, such as transitions to tenofovir/lamivudine/dolutegravir during national scale-up. Medical records and self-report were used to document behavioral factors and tuberculosis preventive therapy, which may have resulted in an overestimation or underestimation. Furthermore, the results may not be entirely applicable to patients starting therapy under more recent national guidelines because many participants started ART during the early stages of Universal Test and Treat implementation, and the observational design prevents causal inference.

**Response to Reviewer 2**

- **General Comments -**

**- To enhance the manuscript’s contribution and clarity, the reviewer recommends the authors address the following points:**

**- General Editorial suggestions -**

**• While the manuscript reads well overall, it would benefit from additional editing and formatting. Specific areas for improvement include:**

**1. Ensure all acronyms are defined at first mention and used consistently thereafter. For example, the “Universal Test and Treat (UTT)” approach is inconsistently referenced with and without the acronym. Other acronyms such as DOT and SGDs appear without explanation (e.g., on page 3 and page 9).**

**Author response:** We thank the reviewer for this valuable comment. In response, all acronyms used in the manuscript were carefully reviewed to ensure clarity and consistency. Each acronym has been clearly defined at its first occurrence in the text and has been used consistently throughout the manuscript. These revisions were made to improve the readability and to avoid any potential ambiguity for the readers.

**2. Correct typographical errors, such as “aer” instead of “are” and “reveled” instead of “revealed” (page 3, last paragraph).**

**Author response:** We sincerely thank the reviewer for carefully identifying the typographical errors and for providing helpful corrections. All the errors specifically pointed out by the reviewer have been corrected as suggested. In addition, we conducted a thorough review of the entire manuscript to identify and correct similar typographical and formatting issues. As a result, all necessary revisions have been implemented to improve the overall clarity, accuracy, and readability of the manuscript.

**3. Clarify sentence structure. For instance, the sentence ending with “…were considered as common predictors associated with new and re-infection of TB” (page 4, first paragraph) does not logically follow the preceding list of factors.**

**Author response:** We thank the reviewer for this insightful suggestion. We have accepted it and corrected the specified issue. In addition, we reviewed and revised the sentence structure throughout the manuscript to address similar problems. The specified paragraph with sentence-structure issues has been revised in the manuscript as follows.

In Ethiopia, pooled estimates from multiple studies indicate that the prevalence of TB/HIV co-infection is approximately 25.59% [21], markedly higher than the national average of 9% [22]. TB is the most frequently detected OI in both pre-ART and on-ART patients [23], and it is also the leading recurrent OIs, comprising 17.5% of cases among HIV patients[24]. Evidence suggests that several factors contribute to new or recurrent TB disease among people living with HIV/AIDS (PLHIV/AIDS), including Cell Differentiation 4 (CD4)lymphocyte count [25-28], Body Mass Index (BMI) [27, 29], behavioral factors such as smoking and alcohol use, as well as sputum smear test results [30-32], and poor adherence to ART and advanced HIV disease [33, 34]. These variables have been commonly identified as predictors of new and recurrent TB.

- **Methods –**

**1. The rationale for calculating the sample size based on “tuberculosis recurrence” is unclear. Why was recurrence used instead of the primary outcome of interest—TB incidence?**

**Author response:** We appreciate the reviewer's crucial comment. At the time of research design, reliable data of tuberculosis incidence in the target group were scarce and extremely variable, making it difficult to calculate a robust sample size solely on incidence. In contrast, TB recurrence among previously treated persons was a well-defined and regularly reported outcome in accessible data and surveillance reports. Because recurrence is a clinically significant and measurable proxy for incident tuberculosis in this high-risk cohort, it was employed to generate a cautious and achievable sample size estimate.

We clarified this rationale in the Methods section, emphasizing that tuberculosis incidence remained the key outcome of interest in analysis and interpretation.

**2. The justification for using a 50% maximum incidence rate due to the “absence of prior studies” seems inconsistent with the literature cited in the discussion, including previous meta-analyses. Please clarify.**

**Author response:**

We appreciate the reviewer’s observation and agree that this point required clarification. The choice of a 50% maximum incidence rate was not intended to imply that there were no earlier studies on tuberculosis incidence in general, but rather that there were no context-specific estimates suitable to our study population, environment, and follow-up duration at the time of sample size computation. Given the uncertainty, we used a conservative assumption of 50%, which maximizes variance while maintaining acceptable statistical power. The meta-analyses provided in the Discussion provide broader, pooled estimates across varied populations and situations; therefore they were not immediately applicable to accurate sample size estimation in our investigation.

**3. Provide clear operational definitions for key study variables, including how TB infection was determined and how independent variables were measured.**

**Author response:** We thank the reviewer for this comment and fully accept it. All necessary operational definitions for both the dependent and independent variables have now been incorporated into the revised manuscript, along with appropriate references.

**Operational definition**

**Censored**: During the follow-up period and at the end of the study, there were no reported cases of tuberculosis infection.

**Event**: diagnosis of TB after initiation of HAART

**Time to incidence of TB:** this is the time from initiation of HAART up to the diagnosis of tuberculosis disease.

**New TB infection:** A TB diagnosis occurring during follow‑up in a patient with no prior history of active TB disease at baseline.

**Re‑infection of Tuberculosis:** A new episode of TB occurring after completion of previous TB treatment (relapse or reinfection), consistent with national TB guidelines defining relapse categories.

**Recurrence**: Recurrent tuberculosis (TB) refers to a new episode of TB diagnosed after a patient has been declared cured or has completed treatment, with cure defined by WHO as negative sputum smear and culture results in the final month of therapy and on at least one previous occasion [41].

**Loss to follow-up:** defined as an ART patient who has not attended any clinic visit in the past three months and is not recorded as deceased or transferred to another HIV care facility.

**Transfer out:** This occurs when a person living with HIV (PLHIV) receiving care at the selected hospitals transfers their treatment to another health facility.

**ART Adherence:**

**Good Adherence** (if adherence greater than 95%, meaning the patient missed fewer than 2 doses out of 30 or fewer than 3 doses out of 60, as documented by ART health personnel);

**Fair Adherence** (if adherence between 85% and 94%, corresponding to 3–5 missed doses out of 30 or 3–9 missed doses out of 60, as recorded by ART staff);

**Poor Adherence** (if adherence below 85%, meaning more than 6 missed doses out of 30 or more than 9 missed doses out of 60, according to ART health personnel documentation) [42].

**Functional status:**

Working: Able to perform usual work in or out of the home;

Ambulatory: Able to perform daily activities but not work;

Bedridden: Not able to perform daily activities, in line with WHO functional status categories. [42].

**Substance use:** Self‑reported current or historical use of substances including alcohol, khat, and cigarettes, as recorded in medical records [43].

**Catchment area:** The geographic area or population served by the hospital where the patient receives care.

**Anemia:** Defined based on WHO criteria as hemoglobin < 12 g/dL for females and < 13 g/dL for males, as recorded on the medical records[44].

**Isoniazid Preventive Therapy (IPT):** Use of daily isoniazid to prevent active TB disease among people living with HIV who do not have active TB symptoms[22].

**Cotrimoxazole Preventive Therapy (CPT):** Use of cotrimoxazole to prevent opportunistic infections among people living with HIV [22].

**Fluconazole Preventive Therapy (FPT):** Use of fluconazole to prevent fungal opportunistic infections in HIV‑positive patients at risk[22].

- **Results -**

**1. On page 8, the manuscript notes the exclusion of 38 patients due to ineligibility. Please specify the eligibility criteria used.**

**Author response**: We thank the reviewer for this reasonable and important question regarding the eligibility criteria and the exclusion of the 38 patients. We would like to clarify that the medical records of these patients were initially eligible for data extraction. As described in the sampling procedure section of the methodology, adult HIV/AIDS patients who initiated ART after the implementation of the Universal Test and Treat strategy were first identified and coded to form the sampling frame. Patients who did not meet the inclusion criteria—specifically, those with incomplete medical records (lacking information on the outcome of interest) and transferred-in patients—were excluded prior to sampling.

From the eligible and coded sampling frame, study participants were then selected using a simple random sampling technique with a computer-generated random number approach. The 38 patients in question were excluded at a later stage, not due to ineligibility of their medical records, but because they did not provide verbal consent. Specifically, these patients either did not respond to phone calls or declined to give verbal consent for the extraction of information from their individual patient folders. Therefore, these cases represent non-respondents rather than ineligible patients.

**2. Table numbering is inconsistent. The results section refers to Tables 1–8, but the attached tables are labeled Tables 9–16. This may be a formatting issue, but the tables should be renumbered accordingly.**

**Author response**: We would like to thank the reviewer for this critical and valuable observation. The comment is correct, and the issue occurred due to the repeated automatic insertion of table captions by the word-processing software. As noted by the reviewer, the results section referred to Tables 1–8, whereas the attached tables were labeled as Tables 9–16. This inconsistency has now been carefully revised and corrected throughout the manuscript in accordance with the reviewer’s recommendation.

**3. There is a discrepancy in the adherence data: the narrative on page 9 reports 770.3% adherence, which is likely a typographical error. Please verify and correct (likely intended to be 70.3%).**

**Author response:** We sincerely thank the reviewer for carefully identifying the typographical errors and for providing helpful corrections. The errors specifically pointed out by the reviewer have been corrected. In addition, a thorough review of the entire manuscript has been done to identify and correct similar issues.

**4. Avoid using the term “substance abuse” if the data only reflect self-reported use. Additionally, clarify which substances were included beyond alcohol, khat, and cigarettes.**

**Author response:** Thank you for the recommendation. We accept it, as the reviewer correctly understands that our aim was to reflect self-reported substance use. In this study, we only considered substances that were already described (alcohol, khat, and cigarettes), and we acknowledge that this may have led to underreporting of other substance use. We have added this as a limitation of the study and have ensured that this clarification is addressed throughout the revised manuscript.

**5. Consider reducing the number of tables and figures. Presenting only the most critical results in the main text and moving others to supplementary materials may improve readability.**

**Authors response:** Thank you for the kind recommendation. In response, we have carefully reviewed all tables and figures and reduced their number by focusing on the most critical and relevant results. Several tables were combined where appropriate to avoid redundancy, and additional non-critical results were moved to the supplementary materials. These changes have improved the clarity and readability of the manuscript, and all revisions are reflected in the revised version.

In response to the reviewers’ recommendation to reduce the number of tables, several tables were merged. Specifically, Tables 2 (“Behavioral characteristics of patients enrolled on HAART with the UTT approach, 2023”), Table 3 (“Clinical and immunological characteristics of patients enrolled on HAART with the UTT approach, 2023”), and Table 4 (“Medication-related characteristics of patients enrolled on HAART with the UTT approach, 2023”) in the previous manuscript version have been compiled into a single table. This consolidated table is now presented as Table 2, titled “Behavioral, Clinical, Immunological, and Medication-Related Characteristics of Patients Enrolled in HAART under the UTT Approach, 2023,” in the revised manuscript.

Additionally, some tables (Table 4: *Log-rank test for equality of survivor functions between categories of covariates*) and figures (Figure 1: *Sampling procedure to assess the incidence of tuberculosis and its predictors among patients on HAART with the UTT approach, 2022*) have been removed from the main manuscript and provided as supplementary material in the Supporting Information, in accordance with the reviewers’ recommendations.

- **Discussion -**

**1. Broad claims in the discussion should be supported by references. For example, on page 14, explanations for gender differences in TB incidence (e.g., social behaviors, hormonal differences) are not cited. Similar unsupported statements appear on pages 15–16.**

**Author response**: Author response: Thank you for this important comment. We agree that broad explanatory statements should be supported by appropriate references. Accordingly, we have revised the Discussion section to include relevant citations supporting explanations for gender differences in tuberculosis incidence, including biological (hormonal and immune response differences), behavioral (smoking, alcohol use, occupational exposure), and health-seeking factors. All previously unsupported statements have now been referenced to strengthen the scientific rigor of the discussion, on the revised version of the manuscript.

**2. On page 15, the statement “Malnutrition, which reduces immunity and promotes tuberculosis activation, could be the source of tuberculosis in HIV patients who are underweight” is misleading. Malnutrition may increase susceptibility but is not a source of TB. Please revise for accuracy.**

**Author response:** Thank you for such a critical and insightful observation, We accept it, and corrected it on the on the revised manuscript as follows.

Underweight HIV patients may have increased catabolic activity, infection, loss of appetite, and decreased intake, which increases the risk of developing TB when compared to persons with a normal BMI. Malnutrition, which reduces immunity and promotes tuberculosis reactivation, could increase susceptibility to tuberculosis in HIV patients who are underweight.

- **References -**

**1. Review reference 20, which includes conflict of interest disclosures for each author. This may not be necessary in the reference list.**

**Author response:** Thank you for such a critical and insightful observation, we accept it, and corrected it on the revised manuscript as follows.

**2. Check all references for broken links.**

**Author response:** We thank the reviewer for this insightful comment. All references were systematically reviewed to identify broken or inactive URLs and DOIs. Necessary corrections and updates were made using official publisher and institutional sources, and the revised links have been incorporated into the revised manuscript.

**3. Ensure acronyms are expanded where appropriate. For example, in reference 22, clarify “FMOH” and specify if it refers to the Ethiopian Federal Ministry of Health.**

**Author response:** We thank the reviewer for this constructive comment. All references were systematically reviewed to ensure that acronyms appearing in the references are fully expanded where appropriate, and the necessary revisions have been incorporated into the revised manuscript.

- **Tables -**

**1. Renumber tables sequentially from Table 1 to Table 8.**

**Authors response:** Thank you for this comment. All tables cited from have now been appropriately renumbered sequentially and included in the results section of the revised manuscript and placed immediately after their respective captions.

**2. Include appropriate footnotes for all tables. For instance, Table 15 (likely intended as Table 2) includes asterisks next to p-values, but no explanation is provided. Also, define all acronyms used in the tables.**

**Author response:** We thank the reviewer for this constructive comment. Appropriate footnotes have now been added to all tables to improve clarity and interpretation. Specifically, the asterisks used to denote levels of statistical significance (e.g., p-values) have been clearly explained in the table footnotes. In addition, all acronyms used in the tables which are not defined on the declaration section have been fully defined in the corresponding footnotes. These revisions have been incorporated throughout the revised manuscript.
